# Tumor regionalization after surgery: Roles of the tumor microenvironment and neutrophil extracellular traps

**DOI:** 10.1038/s12276-022-00784-2

**Published:** 2022-06-28

**Authors:** Su-Bin Kwak, Sang Jin Kim, Jiyoung Kim, Ye-Lim Kang, Chang Woo Ko, Iljin Kim, Jong-Wan Park

**Affiliations:** 1 Department of Pharmacology, Seoul National University College of Medicine, Daehak-ro, Jongno-gu, Seoul, 03080 Korea; 2 Department of Biomedical Science, BK21-plus Education Program, Seoul National University College of Medicine, Daehak-ro, Jongno-gu, Seoul, 03080 Korea; 3 Cancer Research Institute and Ischemic/Hypoxic Disease Institute, Seoul National University College of Medicine, Daehak-ro, Jongno-gu, Seoul, 03080 Korea; 4 Department of Pharmacology, Inha University College of Medicine, Inha-ro, Michuhol-gu, Incheon, 22212 Korea

**Keywords:** Immunoediting, Cancer microenvironment

## Abstract

Surgery is unanimously regarded as the primary strategy to cure solid tumors in the early stages but is not always used in advanced cases. However, tumor surgery must be carefully considered because the risk of metastasis could be increased by the surgical procedure. Tumor surgery may result in a deep wound, which induces many biological responses favoring tumor metastasis. In particular, NETosis, which is the process of forming neutrophil extracellular traps (NETs), has received attention as a risk factor for surgery-induced metastasis. To reduce cancer mortality, researchers have made efforts to prevent secondary metastasis after resection of the primary tumor. From this point of view, a better understanding of surgery-induced metastasis might provide new strategies for more effective and safer surgical approaches. In this paper, recent insights into the surgical effects on metastasis will be reviewed. Moreover, in-depth opinions about the effects of NETs on metastasis will be discussed.

## Cancer treatment: Understanding risk of spread after surgery

Therapies that limit the formation of web-like structures formed by white cells known as neutrophils may lower the risk of cancer spread (metastasis) following surgical tumor removal. Removing solid tumors remains a key cancer treatment, but in some cases surgery itself increases the risk of metastasis. Jong-Wan Park at Seoul National University, South Korea, and co-workers reviewed current understanding of metastasis following surgery. Surgical removal destroys the architecture supporting cancer cells but this can release tumor cells into blood vessels. The stress of deep wounds also affects immune responses, most notably neutrophil extracellular traps (NETs), web-like structures formed by neutrophils to trap and kill pathogens. NETs have previously been implicated in metastasis. In a post-surgical environment enriched in neutrophils and pro-inflammatory cytokines, NET formation may help cancer cells thrive, promoting metastasis.

## Introduction

Surgical resection of tumor masses has been regarded as the primary strategy to effectively eradicate cancer cells. Even in cases of incurable cancers, surgery is often performed to prolong the patient’s lifespan or to relieve cancer pain and some complications associated with tumor masses^1^. However, a wide range of unwanted effects are related to these procedures. In addition to general complications, such as bleeding, thromboembolism, and wound infection, surgery per se can promote cancer metastasis through a series of local and systemic events^2,3^. A growing body of evidence from clinical and experimental studies has suggested that surgery results in a serious wound that disrupts the structural barrier preventing the outspreading of cancer cells, change the properties of the cancer cells and stromal cells remaining in the tumor microenvironment, or impairs the host defense systems against cancers^4–6^. Consequently, these unwanted effects of surgery can trigger the second phase of tumor recurrence and metastasis, which are newly acquired events, rather than just outcomes of incomplete treatment. In particular, infection and inflammation during the postoperative period have been reported to increase the risk of cancer recurrence in patients^7–9^. Notably, these unwanted effects are only found in some patients, not all. Given that the prognostic benefits of surgery are generally greater than the disadvantages, surgical resection should not be abandoned. Nonetheless, it is worthwhile to fully elucidate the mechanisms underlying surgery-triggered aggravation of cancer because it could provide new strategies for more effective and safer surgical approaches for cancers. In this paper, two topics on surgery-triggered cancer metastasis will be discussed. One is a general review concerning the local and systemic influences of cancer surgery on metastasis; the other is the in-depth review concerning the unique role of neutrophils in cancer metastasis.

## Surgery-induced cancer metastasis

**Fig. 1:**
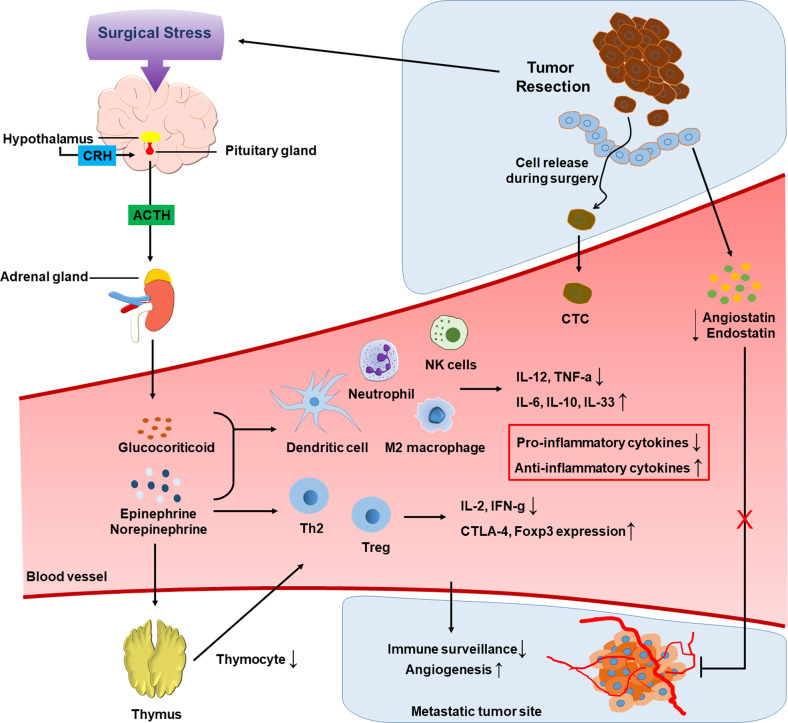
Hypotheses of surgery-triggered cancer metastasis. During tumor resection, tumor cells can be released due to mechanical pressure and vascular injury. Due to surgical stress, the hypothalamic–pituitary–adrenal axis is activated, finally leading to the release of glucocorticoids and catecholamine hormones. These hormones negatively regulate the antitumor activities of innate and adaptive immune cells. Tumor-derived angiogenic inhibitors disappear after removing the primary tumor, which allows metastases to regrow with active neoangiogenesis.

Surgeons have long suspected that surgery, even if it is a necessary step in cancer treatment, facilitates cancer metastasis^3^. This issue remains an unsolved question. Surgery-induced cancer metastasis has been well established in animal models, such as tumor grafts and spontaneous tumorigenesis^10–12^. As many clinical studies have shown an increased incidence of metastasis during the perioperative period, this event also seems to occur in patients with advanced cancers^13,14^. However, whether the surgical resection of primary tumors is beneficial is controversial. A number of perioperative changes, including tumor cell dissemination, tumor-favoring immune responses, and neoangiogenesis, have been proposed to explain surgery-induced metastasis^15,16^. This scenario is summarized in Fig. 1.

## Tumor cell release by surgical injury

As the tumor cells in the vessels can circulate around the body, they are called circulating tumor cells (CTCs). The number of CTCs is generally viewed as a reliable marker indicating poor outcomes for cancer patients because it is closely associated with tumor metastasis^17–24^. Moreover, many lines of evidence suggest that CTCs abruptly increase just after surgery^25–28^. While solid tumors are surgically removed, the tumor architecture is inevitably destroyed due to physical insult, which raises the chance for tumors to shed their cells into the lymphatic and blood vessels. Even externally palpitating tumors for diagnosis could increase the numbers of CTCs in skin cancer and breast cancer^27^. However, how significantly surgery-induced CTC release impacts patient prognosis is still controversial. It is not surprising that the CTC number can increase during the perioperative period. However, the CTC number may be dependent on the tumor volume remaining after surgery. It is eventually reduced after complete resection of CTC-releasing tumors but not after incomplete resection^25^. During surgery, the dissemination and intravasation of tumor cells might be attributed to mechanical pressure and vascular injury^29^. Long after surgery, tumor cells may spread due to increased cell migration and vascular permeability in the altered microenvironment^13,30,31^.

## Immunity altered by surgical stress

Surgical wounds trigger inflammatory responses to favor survival and extravasation of CTCs^28,32,33^. Surgery can disturb the balance between the innate and adaptive immune systems, leading to impaired surveillance of tumors^34,35^. Such an effect on surgery seems to be responsible for the systemic response to stress, including the activation of the hypothalamic–pituitary–adrenal (HPA) axis and subsequent production of stress hormones^36,37^. Upon HPA stimulation, corticotrophin-releasing hormone (CRH) is secreted from the paraventricular nucleus in the hypothalamus and then stimulates the secretion of adrenocorticotropin hormone (ACTH) from the anterior pituitary. The secreted ACTH induces the synthesis and release of glucocorticoids in the adrenal cortex^38^. As excessive glucocorticoids negatively modulate immune functions, immune surveillance against tumors is considered to be impaired by surgical stress, thereby facilitating the growth of tumors remaining around surgical wounds and metastatic tumors at distant sites^39–41^.

In addition to glucocorticoids, during stimulation of the HPA axis, the catecholamine hormones epinephrine and norepinephrine are released from the adrenal medulla^42,43^. Adrenergic receptors (ARs) for these hormones are located on the surface of immune cells. Many studies have shown that α- and β-ARs are both expressed by innate immune cells, including neutrophils, monocytes, macrophages, dendritic cells, and NK cells^44–48^. In particular, β2-AR is an AR subtype expressed at the highest level on both innate and adaptive immune cells. Therefore, β2-AR is regarded as the main mediator responsible for the immune effects of catecholamines^49–52^.

We first review how adrenergic signaling affects innate immunity. β2-AR signaling promotes the M2 differentiation of macrophages, subsequently leading to inhibited production of pro-inflammatory cytokines. In contrast, α-AR signaling has been shown to reverse the effect of β-AR signaling in macrophages^53–58^. In dendritic cells, β2-AR signaling inhibits their differentiation and antigen presentation function. This signaling also suppresses the production of the pro-inflammatory cytokines IL-12 and TNF-α from dendritic cells, whereas it enhances the production of the anti-inflammatory cytokines IL-6, IL-10, and IL-33^49,59–61^. In NK cells, their cytotoxic activity and IFNγ production have been found to be substantially reduced after primary tumors are removed^62,63^. This NK cell suppression may be attributed to increased levels of catecholamines as well as glucocorticoids^64–66^. However, the role of adrenergic signaling in the antitumor activity of NK cells is still controversial because it may differ depending on the type and duration of stress^67–69^. Moreover, there are many reports investigating the adrenergic modulations of other innate immune cells. For example, β2-AR stimulation has been reported to functionally inhibit eosinophils and subsequently aggravate asthma^70^.

In contrast, adrenergic signaling can suppress adaptive immunity. For instance, β-AR signaling decreases the numbers of thymocytes through negative selection in the thymus by activating the p38 signaling pathway^71^. In addition, adrenergic signaling decreases the production of IL-2 and IFN-γ by CD4+ T cells and prevents their proliferation. This signaling also regulates Th1 and Th2 differentiation. β2-AR activation was found to promote CD4+ T-cell polarization toward a Th2 phenotype^72–76^. In memory and effector CD8+ T cells, which express β2-AR at a higher level than naive T cells, β2-AR signaling downregulates IL-2 and IFN-γ expression under stimulation^77,78^. In regulatory T cells (Tregs), however, β2-AR signaling enhances immune-suppressive activity by inducing CTLA-4 and Foxp3 expression^72,79^. The in-depth discussion about this subject is omitted because it is beyond the scope of this review.

## Angiogenic state altered by removal of primary tumors

A striking hypothesis about angiogenic competition among tumors was experimentally tested by Dr. Folkman and his colleagues^80^. In mice bearing a primary tumor, it was observed that the removal of the primary tumor facilitated the growth of highly vascularized metastases. For hypothetical mechanisms, primary tumors may secrete angiogenic inhibitors as well as angiogenic activators. In the microenvironment of the primary tumor, the activators are abundant enough to overwhelm the inhibitors. In the systemic circulation, however, the activators quickly decay, whereas the inhibitors remain stable. Consequently, small metastases at distant sites are affected to a greater extent by angiogenic inhibitors than by activators, leading to the dormancy of metastases due to limited vascularization. After the primary tumor is surgically removed, the metastases can start to grow vigorously via neoangiogenesis because the circulating inhibitors disappear. Angiostatin and endostatin are regarded as representatives of tumor-derived angiogenesis inhibitors^81–83^.

## Roles of netosis in surgery-induced cancer metastasis

Neutrophils are the most abundant type of granulocytes, comprising 40–70% of all white blood cells. These cells neutralize invading microorganisms and act as the main mediators of inflammation. They rapidly accumulate in inflamed areas, where they undergo highly diverse reactions^84–87^. These cells not only play defensive roles against harmful microorganisms but also clear dead cells for tissue regeneration^88–90^. However, since neutrophils also play harmful roles in many inflammation-associated diseases, they are considered a double-edged sword^91^. In addition, neutrophils play various roles in the initiation and progression of cancer^92–94^. In particular, a unique phenomenon named NETosis has been intensively investigated as a pathogenic event in many inflammatory and neoplastic diseases^95–101^. NETosis refers to the formation of neutrophil extracellular traps (NETs), which are large extracellular complexes composed of chromatin and cytoplasmic/granular proteins^102,103^. Recently, NETosis has been highlighted as an inflammatory event that promotes cancer metastasis^104,105^.

## NETs and NETosis

Neutrophil extracellular traps (NETs), which were first identified by Volker–Brinkmann and Arturo–Zychlinsky, are fishing net-like structures that can entrap microorganisms invading blood and tissues^86^. Once activated, neutrophils produce intracellular precursors by using DNA, histones, and granular and cytoplasmic proteins and then spread the mature form of NETs out around themselves. A series of these events is called NETosis. In NETs, the following proteins are included: neutrophil elastase, myeloperoxidase, cathepsin G, proteinase 3, lactoferrin, gelatinase, lysozyme C, calprotectin, neutrophil defensins, and cathelicidins^106–110^. As NETs contain a high content of DNA threads, they are sticky enough to entrap and immobilize microorganisms and then kill them using lethal enzymes. Therefore, NETosis is currently defined as an innate immune response against infection. In addition to its antimicrobial activity, NETosis plays a pivotal role in noninfectious autoimmune diseases, such as systemic lupus erythematosus, rheumatoid arthritis, and psoriasis^96,99,111^. Moreover, NETosis is involved in other inflammatory diseases, such as vasculitis, intravascular thrombosis, atherosclerosis, periodontitis, and diabetes^112–116^.

Although the precise mechanisms underlying NETosis are still being investigated, some of the molecular pathways have been identified in two types of NEToses, suicidal (or lytic) NETosis and vital NETosis^117^. Suicidal NETosis mainly depends on the production of reactive oxygen species (ROS)^102^. Many stimuli, such as phorbol 12-myristate 13-acetate (PMA), bacterial endotoxins, and interleukins, have been found to induce suicidal NETosis. Indeed, PMA and IL-8 are widely used to induce suicidal NETosis in vitro^86,102^. Alternatively, suicidal NETosis can be induced by antibodies (especially the Fc region) binding to specific receptors on the surface of neutrophils^118^. Following the stimulation of neutrophils, protein kinase C (PKC) is activated via diverse pathways and in turn activates the Raf-MEK-ERK signaling cascades, followed by ROS production from NADPH oxidases^119,120^. Alternatively, IL-8 enhances the cytoplasmic levels of ROS, which are mediated by the following process: NF-κB activation via the CXCR2-PI3K-AKT pathway and iNOS and COX2 induction by NF-κB^121–123^. Then, ROS play central roles in suicidal NETosis because they induce the release of the serine protease neutrophil elastase (NE) and myeloperoxidase (MPO) from azurophilic granules and activate peptidyl arginine deiminase 4 (PAD4). PAD4 citrullinates histones in the presence of calcium ions, leading to chromatin decondensation^124,125^. After the nucleus becomes deformed and ruptured, the decondensed chromatin is released to the cytoplasm and entangled with proteolytic enzymes and other proteins, which are the intracellular precursors of NETs^126^. Finally, the intracellular NET complexes are released through a broken part of the plasma membrane. Since neutrophils die during this process, it is called suicidal NETosis.

**Fig. 2:**
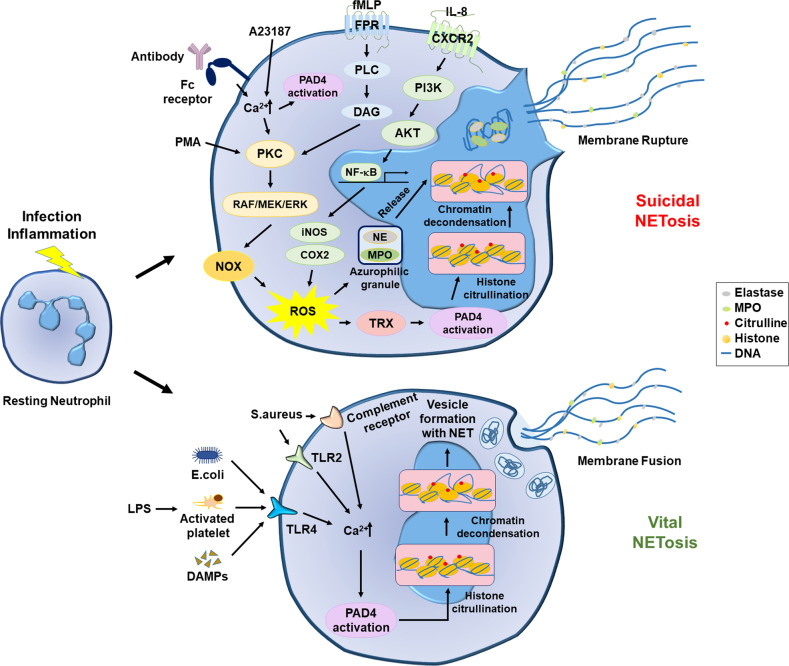
The molecular mechanisms underlying suicidal NETosis and vital NETosis. Suicidal NETosis (top). PKC and ROS play pivotal roles in suicidal NETosis. PKC can be activated directly by PMA or indirectly by A23187 and antibodies that increase Ca^2+^ in the cytoplasm. PKC can also be activated through the FPR-PLC-DAG signaling pathway. Activated PKC facilitates NOX-driven ROS production through the RAF/MEK/ERK pathway. ROS production can be stimulated by IL-8, which activates NF-κB and in turn induces the production of iNOS and COX2 by ROS. Subsequently, ROS induces the release of neutrophil enzymes from azurophilic granules and activates PAD4 by inducing thioredoxin (TRX). Ca^2+^ also contributes to the activation of PAD4. Activated PAD4 catalyzes the citrullination of histones, leading to chromatin decondensation. Finally, the chromatin threads and neutrophil enzymes form NETs in the cytoplasm, and neutrophils release NETs through membrane rupture. Vital NETosis (bottom). This process is mainly mediated by Ca^2+^ but is independent of ROS. The cytoplasmic level of Ca^2+^ can be elevated through diverse pathways, such as TLR2, TLT4, and the complement receptor. The Ca^2+^ elevation stimulates PAD4, which sequentially provokes histone citrullination, histone decondensation, and NET formation. As NETs are stored within vesicles, neutrophils can release them without membrane rupture.

However, there is another type of NETosis called vital NETosis. Compared with suicidal NETosis, vital NETosis occurs independently of ROS production from NADPH oxidase^127^. Vital NETosis can be induced by Gram-negative bacteria. LPS in the outer membrane of the bacteria interacts with Toll-like receptor 2 (TLR2) on neutrophils and TLR4 on the surface of platelets, which triggers NETosis^128^. The stimulation of these receptors increases the cytoplasmic level of Ca^2+^ and subsequently stimulates PAD4, leading to NET formation. As NETs are entrapped within vesicles, neutrophils can release them without membrane rupture. The neutrophils undergoing this type of NETosis are still alive, so this process is called vital NETosis. However, the signaling pathway responsible for vital NETosis remains unclear. The molecular mechanisms underlying NETosis are summarized in Fig. 2.

## NETosis in the tumor microenvironment

Experimental and clinical studies have revealed that NETs are present in a variety of cancers, such as lung cancer, colon cancer, ovarian cancer, and leukemia^129–133^. This finding suggests that neutrophils actively undergo NETosis in the tumor microenvironment. To date, several hypotheses have been proposed to explain why NETosis is stimulated within tumors. Hypoxia could be one of the reasons because it often develops in growing solid tumors and robustly induces the transcription factor HIF-1. Indeed, McInturff et al. (2012) demonstrated that HIF-1 in neutrophils plays a critical role in NETosis and bacteria-killing activity^134^. Some cytokines enriched in the tumor microenvironment may stimulate tumor-infiltrating neutrophils to undergo NETosis. For instance, the pro-inflammatory cytokines IL-8, IL-17, G-CSF, CXCL5, and CXCL6 are released from tumor cells and recruit neutrophils in the bone marrow to tumor regions^135–140^. Moreover, many recent reports have suggested that most cytokines potentially initiate or facilitate NETosis in vitro or in vivo^141–143^. In addition to cytokines, some tumor-derived proteases and tumor exosomes have been reported to induce NETosis^144,145^. Therefore, NETosis generally actively progresses in the tumor microenvironment.

Recently, NETosis has been investigated as an emerging surrogate marker for cancer diagnosis. The plasma levels of NETs were found to be higher in patients with several types of tumors, including lung cancer, pancreatic cancer, and bladder cancer, than in healthy controls^146^. In lung cancer patients, NETs are present in lung tissues and are also detected in peripheral blood and sputum^130^. In the case of colon cancer, neutrophils from cancer patients were found to undergo NETosis at a higher level under in vitro stimulation than those from healthy controls^147^. Interestingly, such in vitro results positively correlated with the poor clinical outcomes of the patients. Moreover, immunofluorescence staining showed that some components of NETs were detected at the highest level in metastases originating from breast and colon cancers^148^. Recently, a clinical study demonstrated that the serum levels of NET components at the preoperative stage were associated with poor survival in cancer patients^149^. Given these reports, the plasma levels of NET components could be emerging biomarkers to predict the clinical outcomes of cancer patients.

To date, NETosis can be estimated by detecting molecular markers, such as extracellular DNA, citrullinated histone 3 (Cit-H3), and neutrophil-derived protein complexes. However, most researchers tend to confirm this process using more than two markers because each marker has low reliability. Nonetheless, immunochemistry using an anti-Cit-H3 antibody is regarded as a standard method because H3 citrullination is very specific to NETosis. However, even Cit-H3 immunostaining has some drawbacks in use because the procedure is difficult, time-consuming, and not applicable to real-time monitoring. Recently, a research team successfully developed a new NET detection method using the extracellular DNA-intercalating dye CDr15^150^. This method was found to be easier than previous methods and applicable to the real-time tracing of NETosis. Considering the clinical importance of NETosis, it is worthwhile to develop new NET-detecting materials that are applicable to experimental and clinical studies.

## Neutrophil activation and NETosis following surgical injury

Surgical trauma and subsequent complications, including wound infection, increase neutrophil counts in the peripheral blood through increased granulopoiesis in the bone marrow^151–154^. Additionally, tissue injury affects the function of circulating neutrophils^155,156^. The changes in neutrophil functions depend on the micromilieu of the damaged tissue. In wounds, damaged and necrotic cells express the signals responsible for early neutrophil recruitment, which are damage-associated molecular patterns (DAMPs)^157–160^. DAMPs are cellular components including DNA, histones, ATP, interleukin-1α, high mobility group protein B1 (HMGB1), N-formyl peptides, and others. DAMPs directly activate neutrophils through G-protein-coupled receptors^161,162^. Indirectly, DAMPs can stimulate surrounding tissues to produce chemokines and lipid mediators for neutrophil chemotaxis^163,164^. In wounds, neutrophils clear necrotic cells and invading microorganisms^165–167^. Moreover, they release various cytokines responsible for tissue repair^168,169^. Indeed, neutrophils express and store a variety of growth factors and angiogenic factors that contribute to regeneration and revascularization^170,171^. For example, the proliferative cytokines TGFβ and IL-10 and the angiogenic factor VEGF are representative of neutrophil-derived tissue repair proteins.

As mentioned above, NETosis is a defense system to protect the body from invading pathogens^172^. However, when neutrophils are excessively stimulated, they produce excess NETs, thereby leading to pathological consequences^173,174^. To eliminate a visceral tumor, surgeons make a deep incision on the skin, remove a part of the tumor-bearing organ, and reconstruct surrounding tissues for functional recovery. Since such a procedure involves large wounds, it can induce severe inflammation and subsequently stimulate neutrophils to undergo NETosis. Given many clinical reports showing that the plasma levels of NETosis markers are elevated after major surgeries, NETs formed at surgical wounds are believed to circulate throughout the body along with blood and lymphatic streams^175–178^. Since NETs circulate in the peripheral blood, the components of NETs could be surrogate markers for evaluating the progression of cancers in clinical settings^179–182^.

## Roles of NETs in tumor metastasis

**Fig. 3:**
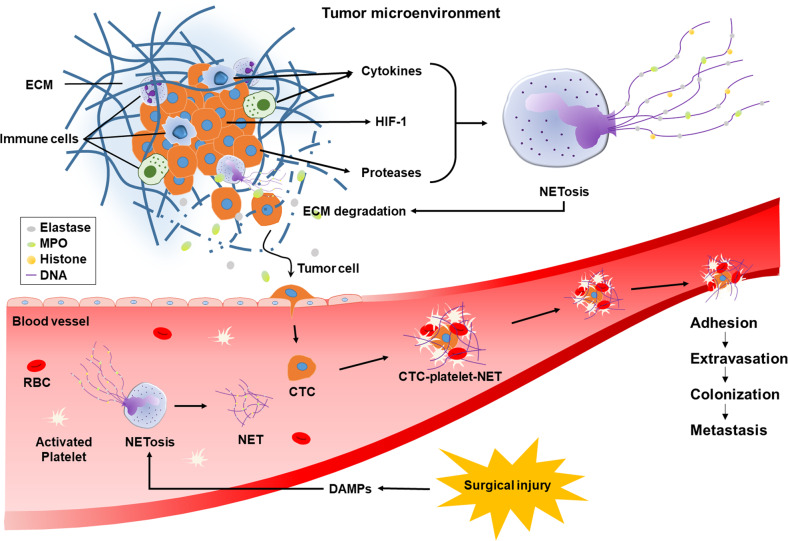
Hypothetical roles of NETs in surgery-triggered cancer metastasis. In the tumor microenvironment, NETosis is stimulated by various cytokines, proteases, and hypoxia-induced HIF-1. Proteolytic enzymes in NETs loosen the ECM and capillary wall to promote the intravasation of cancer cells. Neutrophils undergo NETosis, and platelets are activated by surgical DAMPs. NETs and platelets wrap CTCs, which protects them from attack by immune cells and shearing force by blood flow. NET-based aggregates can be attached to the capillary wall, where tumor cells move out and form new metastatic colonies.

Metastasis refers to tumor cell spread out of the original location and the formation of secondary lesions at distant sites. Tumor cells metastasize through the following sequences: local invasion, intravasation into the blood or lymphatic vessels, escape from the immune system, anchoring to capillaries in target organs, extravasation into the organs, transformation from dormant cells to proliferating cells, colonization to micrometastases, and growth to macrometastases^183–189^. Many lines of evidence suggest that NETs promote metastasis at multiple steps, which are summarized in Fig. 3.

NETs promote the local invasion of cancer cells by degrading the extracellular matrix (ECM)^190,191^. ECMs, which are mainly composed of fibrous proteins and polysaccharides, act as a barrier blocking cell movement, and thus, degrading the ECM is essential for tumor cell invasion^192^. As mentioned previously, NETs contain many proteolytic enzymes, such as neutrophil elastase, matrix metalloproteinase 9, and cathepsin G. These NET enzymes loosen the ECMs to allow cancer cell movement. NETs also promote the intravasation of cancer cells by increasing vascular permeability^193^. The wall of a capillary consists of two layers: the endothelial layer and the outer basement membrane (mainly made of connective tissues). Capillaries (especially, continuous capillaries) are characterized by a complete endothelial lining with tight junctions between endothelial cells. The tight junction is impermeable to macromolecules but allows the passage of small molecules, such as water, ions, gases, metabolites, and hormones. Therefore, cancer cells should penetrate the two layers to enter the circulation, which cannot occur normally. Given that the basement membrane and the tight junction are both mainly composed of proteins, it is not surprising that protease-containing NETs loosen the vascular wall and allow cancer cells to penetrate into the vessel. Moreover, pro-inflammatory cytokines released from activated neutrophils escalate the enhancement of vascular permeability^194^.

Even if cancer cells successfully enter the circulation, only a few of them can survive to finally establish metastases. Indeed, millions of tumor cells are released into the circulation every day, but metastases are not observed as frequently as we estimate. There are two obstacles that circulating tumor cells (CTCs) need to overcome: the shearing force by blood flow and the immune system. NETs can wrap up CTCs with platelets owing to their sticky jelly-like properties^195^. By doing so, NETs can act as armor to protect CTCs from the shearing force and the attack of immune cells. Najmeh et al. also suggested that β1-integrin plays an important role in the interaction between CTCs and NETs^196^. In case of tumor surgery, neutrophils and platelets can be simultaneously activated, which facilitates the formation of NET-platelet-CTC aggregates.

Finally, the CTC aggregates stop traveling to some capillaries in distant organs because they are too sticky and large to continuously circulate. The CTC aggregates are entrapped in or adhered to the inside walls of capillaries and in turn extravasate into the parenchyma to establish new colonies. The termination of this journey may be a new focus of metastasis. In the growth of micrometastases, the involvement of NETs has been demonstrated in several animal studies. For instance, when tumor-bearing mice were injected with DNase (decomposing NETs), the growth of the preexisting metastases was significantly retarded^180^. In mice subjected to abdominal surgery, NETs in the peritoneum collected tumor cells and provided a microenvironment favoring tumor survival and growth. These results suggest that NETosis is a potential target to prevent surgery-induced tumor metastasis.

After metastasizing to distant tissues, tumor cells are often found to remain dormant for a period of time and unexpectedly regrow later^197^. To date, little is known about the molecular mechanisms underlying tumor dormancy and reactivation. In addition to the metastatic process, NETs are believed to participate in the reactivation of dormant cancer cells in metastatic regions^198^. An animal study showed that dormant micrometastases became aggressively growing metastases after lung inflammation was induced by tobacco smoke or nasal instillation of lipopolysaccharide. In this study, the NET-associated proteases NE and MMP-9 were found to be responsible for the reactivation of dormant cancer cells. Laminin cleavage by enzymes may induce reactivation through the integrin α3β1 signaling pathway. However, the effects of NETs on the reactivation of dormant cells remain uncertain.

## Conclusion

Here, we discuss how distant metastasis paradoxically increases after resection of the primary tumor. From two points of view, this topic was discussed: one is a general review concerning surgery-triggered metastasis; the other is a review concerning the roles of NETs in metastasis. After surgical removal of tumors, the tumors and surrounding tissues are harshly handled, which increases the chance for tumor cells to spread. Because the surgery results in a deep wound, it might provoke systemic stress, which inhibits immunity against tumor cells. Moreover, as small metastases can escape from the angiogenic control of the primary tumor, they can grow vigorously after tumor surgery and are clinically detected as multiple metastases. Second, we discuss recent advances in tumor- and surgery-induced NETosis. NETosis is an innate immune process that entraps and kills microorganisms, but it also plays a pathogenic role in many inflammatory diseases. Neutrophils are activated and subsequently undergo NETosis in the tumor microenvironment and the surgical wound, both of which are enriched with pro-inflammatory cytokines. Furthermore, many reports have suggested that NETs stimulate the entire metastatic process from local invasion of cancer cells to colonization/growth. Therefore, NETosis could be an emerging target for blocking tumor metastasis after tumor surgery.

In fact, surgery is a very complicated procedure because it is accompanied by many medications for anesthesia, analgesia, muscle relaxation, and infection control. Obviously, these medications during the perioperative period should be considered risk factors that may affect tumor metastasis. Nonetheless, we restrictively focused on the effects of surgical procedures and wounds on tumor cell spreading and metastasizing to distant organs. Considering that surgery-related medications are doctors’ most likely options, we can try to substitute safer drugs for metastasis-inducing drugs. We hope that this topic will be discussed in future studies.

## References

[1] Wyld L, Audisio RA, Poston GJ (2015). The evolution of cancer surgery and future perspectives. Nat. Rev. Clin. Oncol..

[2] Demicheli R, Retsky MW, Hrushesky WJ, Baum M, Gukas ID (2008). The effects of surgery on tumor growth: a century of investigations. Ann. Oncol..

[3] Tohme S, Simmons RL, Tsung A (2017). Surgery for cancer: a trigger for metastases. Cancer Res..

[4] Alieva M, van Rheenen J, Broekman MLD (2018). Potential impact of invasive surgical procedures on primary tumor growth and metastasis. Clin. Exp. Metastasis..

[5] Chen, Z. et al. Surgical stress and cancer progression: the twisted tango. *Mol. Cancer*10.1186/s12943-019-1058-3 (2019). 10.1186/s12943-019-1058-3 PMC6717988 31477121

[6] Tang F, Tie Y, Tu C, Wei X (2020). Surgical trauma-induced immunosuppression in cancer: recent advances and the potential therapies. Clin. Transl. Med..

[7] Nojiri T (2017). Long-term impact of postoperative complications on cancer recurrence following lung cancer surgery. Ann. Surg. Oncol..

[8] Matsubara D (2020). The impact of postoperative inflammation on recurrence in patients with colorectal cancer. Int. J. Clin. Oncol..

[9] Tsujimoto H (2021). Impact of postoperative infectious complications on adjuvant chemotherapy administration after gastrectomy for advanced gastric cancer. Jpn. J. Clin. Oncol..

[10] Zhang Y, Zhang N, Hoffman RM, Zhao M (2015). Surgically-induced multi-organ metastasis in an orthotopic syngeneic imageable model of 4T1 murine breast cancer. Anticancer Res..

[11] Tsuchiya Y (2003). Increased surgical stress promotes tumor metastasis. Surgery.

[12] Al-Sahaf O, Wang JH, Browne TJ, Cotter TG, Redmond HP (2010). Surgical injury enhances the expression of genes that mediate breast cancer metastasis to the lung. Ann. Surg..

[13] Alieva, M. et al. Preventing inflammation inhibits biopsy-mediated changes in tumor cell behavior. *Sci. Rep*. 10.1038/s41598-017-07660-4 (2017). 10.1038/s41598-017-07660-4 PMC5548904 28790339

[14] Peeters CF, De Waal RM, Wobbes T, Westphal JR, Ruers TJ (2006). Outgrowth of human liver metastases after resection of the primary colorectal tumor: a shift in the balance between apoptosis and proliferation. Int. J. Cancer.

[15] Horowitz M, Neeman E, Sharon E, Ben-Eliyahu S (2015). Exploiting the critical perioperative period to improve long-term cancer outcomes. Nat. Rev. Clin. Oncol..

[16] Neeman E, Ben-Eliyahu S (2013). Surgery and stress promote cancer metastasis: new outlooks on perioperative mediating mechanisms and immune involvement. Brain Behav. Immun..

[17] Lucci A (2012). Circulating tumour cells in non-metastatic breast cancer: a prospective study. Lancet Oncol..

[18] Hofman V (2011). Preoperative circulating tumor cell detection using the isolation by size of epithelial tumor cell method for patients with lung cancer is a new prognostic biomarker. Clin. Cancer Res..

[19] Cohen S (2009). Prognostic significance of circulating tumor cells in patients with metastatic colorectal cancer. Ann. Oncol..

[20] Pantel K, Brakenhoff RH, Brandt B (2008). Detection, clinical relevance and specific biological properties of disseminating tumour cells. Nat. Rev. Cancer.

[21] Paterlini-Brechot P, Benali NL (2007). Circulating tumor cells (CTC) detection: clinical impact and future directions. Cancer Lett..

[22] Nagrath S (2007). Isolation of rare circulating tumour cells in cancer patients by microchip technology. Nature.

[23] Cristofanilli M (2005). Circulating tumor cells: a novel prognostic factor for newly diagnosed metastatic breast cancer. J. Clin. Oncol..

[24] Cristofanilli M (2004). Circulating tumor cells, disease progression, and survival in metastatic breast cancer. N. Engl. J. Med..

[25] Juratli, M. A. et al. In vivo long-term monitoring of circulating tumor cells fluctuation during medical interventions. *PLoS One*10.1371/journal.pone.0137613 (2015). 10.1371/journal.pone.0137613 PMC4569172 26367280

[26] Mathenge EG (2014). Core needle biopsy of breast cancer tumors increases distant metastases in a mouse model. Neoplasia.

[27] Juratli MA (2014). Real‐time monitoring of circulating tumor cell release during tumor manipulation using in vivo photoacoustic and fluorescent flow cytometry. Head. Neck.

[28] Yu, J.-j. et al. Effect of surgical liver resection on circulating tumor cells in patients with hepatocellular carcinoma. *BMC cancer*10.1186/s12885-018-4744-4 (2018). 10.1186/s12885-018-4744-4 PMC6102841 30126375

[29] Bockhorn M, Jain RK, Munn LL (2007). Active versus passive mechanisms in metastasis: do cancer cells crawl into vessels, or are they pushed?. Lancet Oncol..

[30] Harney AS (2015). Real-time imaging reveals local, transient vascular permeability, and tumor cell intravasation stimulated by TIE2hi macrophage–derived VEGFA. Cancer Discov..

[31] Maniwa Y, Okada M, Ishii N, Kiyooka K (1998). Vascular endothelial growth factor increased by pulmonary surgery accelerates the growth of micrometastases in metastatic lung cancer. Chest.

[32] Li, S. et al. Less micrometastatic risk related to circulating tumor cells after endoscopic breast cancer surgery compared to open surgery. *BMC Cancer*10.1186/s12885-019-6158-3 (2019). 10.1186/s12885-019-6158-3 PMC6842272 31703643

[33] Sawabata N (2020). Circulating tumor cells detected only after surgery for non-small cell lung cancer: is it a predictor of recurrence?. J. Thorac. Dis..

[34] Angele MK, Chaudry IH (2005). Surgical trauma and immunosuppression: pathophysiology and potential immunomodulatory approaches. Langenbecks Arch. Surg..

[35] Lin E, Calvano SE, Lowry SF (2000). Inflammatory cytokines and cell response in surgery. Surgery.

[36] Gibbison B, Angelini GD, Lightman SL (2013). Dynamic output and control of the hypothalamic-pituitary-adrenal axis in critical illness and major surgery. Br. J. Anaesth..

[37] DeKeyser FG, Leker RR, Weidenfeld J (2000). Activation of the adrenocortical axis by surgical stress: involvement of central norepinephrine and interleukin-1. Neuroimmunomodulation.

[38] Gibbison B (2015). Dynamic pituitary-adrenal interactions in response to cardiac surgery. Crit. Care Med..

[39] Brokaw JJ (1998). Glucocorticoid-induced apoptosis of dendritic cells in the rat tracheal mucosa. Am. J. Respir. Cell Mol. Biol..

[40] Piemonti L (1999). Glucocorticoids affect human dendritic cell differentiation and maturation. J. Immunol..

[41] Rea D (2000). Glucocorticoids transform CD40-triggering of dendritic cells into an alternative activation pathway resulting in antigen-presenting cells that secrete IL-10. Blood.

[42] McCorry, L. K. Physiology of the autonomic nervous system. *Am. J. Pharm. Educ*. 10.5688/aj710478 (2007). 10.5688/aj710478 PMC1959222 17786266

[43] Sun N (2018). High-resolution tissue mass spectrometry imaging reveals a refined functional anatomy of the human adult adrenal gland. Endocrinology.

[44] Nance DM, Sanders VM (2007). Autonomic innervation and regulation of the immune system (1987-2007). Brain Behav. Immun..

[45] Ordovas-Montanes J (2015). The regulation of immunological processes by peripheral neurons in homeostasis and disease. Trends Immunol..

[46] Cosentino M (1999). Endogenous catecholamine synthesis, metabolism, storage and uptake in human neutrophils. Life Sci..

[47] Elenkov IJ, Wilder RL, Chrousos GP, Vizi ES (2000). The sympathetic nerve–an integrative interface between two supersystems: the brain and the immune system. Pharmacol. Rev..

[48] Straub RH (2004). Complexity of the bi-directional neuroimmune junction in the spleen. Trends Pharmacol. Sci..

[49] Herve J (2013). β2-Adrenoreceptor agonist inhibits antigen cross-presentation by dendritic cells. J. Immunol..

[50] Sanders VM (2012). The beta2-adrenergic receptor on T and B lymphocytes: do we understand it yet?. Brain Behav. Immun..

[51] Maestroni GJ, Mazzola P (2003). Langerhans cells β2-adrenoceptors: role in migration, cytokine production, Th priming and contact hypersensitivity. J. Neuroimmunol..

[52] Salicru, A. N. et al. Variable effects of combination corticosteroids and catecholamines on total beta 2-adrenergic receptors in human CD4+T-lymphocytes. *J. Allergy Clin. Immun*. 10.1016/j.jaci.2006.11.585 (2007).

[53] Tang L (2015). Sympathetic nerve activity maintains an anti-inflammatory state in adipose tissue in male mice by inhibiting TNF-alpha gene expression in macrophages. Endocrinology.

[54] Luan B (2014). Leptin-mediated increases in catecholamine signaling reduce adipose tissue inflammation via activation of macrophage HDAC4. Cell Metab..

[55] Grailer JJ, Haggadone MD, Sarma JV, Zetoune FS, Ward PA (2014). Induction of M2 regulatory macrophages through the β2-adrenergic receptor with protection during endotoxemia and acute lung injury. J. Innate Immun..

[56] Garcia JJ, del Carmen Saez M, De la Fuente M, Ortega E (2003). Regulation of phagocytic process of macrophages by noradrenaline and its end metabolite 4-hydroxy-3-metoxyphenyl-glycol. Role of α- and β-adrenoreceptors. Mol. Cell. Biochem..

[57] Qin JF (2015). Adrenergic receptor β2 activation by stress promotes breast cancer progression through macrophages M2 polarization in tumor microenvironment. BMB Rep..

[58] Sloan EK (2010). The sympathetic nervous system induces a metastatic switch in primary breast cancer. Cancer Res..

[59] Agac D, Estrada LD, Maples R, Hooper LV, Farrar JD (2018). The β2-adrenergic receptor controls inflammation by driving rapid IL-10 secretion. Brain Behav. Immun..

[60] Chen C (2012). β-Adrenergic receptors stimulate interleukin-6 production through Epac-dependent activation of PKCdelta/p38 MAPK signalling in neonatal mouse cardiac fibroblasts. Br. J. Pharmacol..

[61] Yanagawa Y, Matsumoto M, Togashi H (2011). Adrenoceptor-mediated enhancement of interleukin-33 production by dendritic cells. Brain Behav. Immun..

[62] Angka L (2018). Natural killer cell IFNgamma secretion is profoundly suppressed following colorectal cancer surgery. Ann. Surg. Oncol..

[63] Reinhardt, R. et al. Invasive surgery impairs the regulatory function of human CD56 bright natural killer cells in response to Staphylococcus aureus. Suppression of interferon-gamma synthesis. *PLoS One*10.1371/journal.pone.0130155 (2015). 10.1371/journal.pone.0130155 PMC4474941 26090673

[64] Nair MP, Schwartz SA (1984). Immunomodulatory effects of corticosteroids on natural killer and antibody-dependent cellular cytotoxic activities of human lymphocytes. J. Immunol..

[65] Holbrook NJ, Cox WI, Horner HC (1983). Direct suppression of natural killer activity in human peripheral blood leukocyte cultures by glucocorticoids and its modulation by interferon. Cancer Res..

[66] Chen L, Jondal M, Yakimchuk K (2018). Regulatory effects of dexamethasone on NK and T cell immunity. Inflammopharmacology.

[67] Page GG, Ben-Eliyahu S (2000). Natural killer cell activity and resistance to tumor metastasis in prepubescent rats: deficient baselines, but invulnerability to stress and beta-adrenergic stimulation. Neuroimmunomodulation.

[68] Shakhar G, Ben-Eliyahu S (1998). In vivo β-adrenergic stimulation suppresses natural killer activity and compromises resistance to tumor metastasis in rats. J. Immunol..

[69] Inbar, S. et al. Do stress responses promote leukemia progression? an animal study suggesting a role for epinephrine and prostaglandin-E2 through reduced NK activity. *PLoS One*10.1371/journal.pone.0019246 (2011). 10.1371/journal.pone.0019246 PMC3084788 21559428

[70] Zhou X, Dang YJ, Wang GF, Jin XQ (2017). Effects of Aspergillus fumigatus on glucocorticoid receptor and β2-adrenergic receptor expression in a rat model of asthma. Exp. Lung Res..

[71] Lajevic MD, Suleiman S, Cohen RL, Chambers DA (2011). Activation of p38 mitogen-activated protein kinase by norepinephrine in T-lineage cells. Immunology.

[72] Guereschi MG (2013). Beta2-adrenergic receptor signaling in CD4+ Foxp3+ regulatory T cells enhances their suppressive function in a PKA-dependent manner. Eur. J. Immunol..

[73] Kim BJ, Jones HP (2010). Epinephrine-primed murine bone marrow-derived dendritic cells facilitate production of IL-17A and IL-4 but not IFN-gamma by CD4+ T cells. Brain Behav. Immun..

[74] Pilipovic I (2019). Noradrenaline modulates CD4+ T cell priming in rat experimental autoimmune encephalomyelitis: a role for the α_1_-adrenoceptor. Immunol. Res..

[75] McAlees JW (2011). Epigenetic regulation of beta2-adrenergic receptor expression in T(H)1 and T(H)2 cells. Brain Behav. Immun..

[76] Loza MJ, Peters SP, Foster S, Khan IU, Penn RB (2007). beta-Agonist enhances type 2 T-cell survival and accumulation. J. Allergy Clin. Immunol..

[77] Estrada LD, Agac D, Farrar JD (2016). Sympathetic neural signaling via the beta2-adrenergic receptor suppresses T-cell receptor-mediated human and mouse CD8(+) T-cell effector function. Eur. J. Immunol..

[78] Strell, C. et al. Divergent effects of norepinephrine, dopamine and substance P on the activation, differentiation and effector functions of human cytotoxic T lymphocytes. *BMC Immunol*. 10.1186/1471-2172-10-62 (2009). 10.1186/1471-2172-10-62 PMC2794263 19968887

[79] Zhou L (2016). Propranolol attenuates surgical stress-induced elevation of the regulatory T cell response in patients undergoing radical mastectomy. J. Immunol..

[80] Hahnfeldt P, Panigrahy D, Folkman J, Hlatky L (1999). Tumor development under angiogenic signaling: a dynamical theory of tumor growth, treatment response, and postvascular dormancy. Cancer Res..

[81] O’reilly M (1997). Angiostatin: an endogenous inhibitor of angiogenesis and of tumor growth. Regul. angiogenesis.

[82] O’Reilly MS (1997). Endostatin: an endogenous inhibitor of angiogenesis and tumor growth. Cell.

[83] Holmgren L, O’Reilly MS, Folkman J (1995). Dormancy of micrometastases: balanced proliferation and apoptosis in the presence of angiogenesis suppression. Nat. Med..

[84] Phillipson M, Kubes P (2011). The neutrophil in vascular inflammation. Nat. Med..

[85] Ley K, Laudanna C, Cybulsky MI, Nourshargh S (2007). Getting to the site of inflammation: the leukocyte adhesion cascade updated. Nat. Rev. Immunol..

[86] Brinkmann V (2004). Neutrophil extracellular traps kill bacteria. Science.

[87] Mantovani A, Cassatella MA, Costantini C, Jaillon S (2011). Neutrophils in the activation and regulation of innate and adaptive immunity. Nat. Rev. Immunol..

[88] Yang, W. T. et al. Neutrophils promote the development of reparative macrophages mediated by ROS to orchestrate liver repair. *Nat. Commun*. 10.1038/s41467-019-09046-8 (2019). 10.1038/s41467-019-09046-8 PMC6403250 30842418

[89] Kuhl AA (2007). Aggravation of different types of experimental colitis by depletion or adhesion blockade of neutrophils. Gastroenterology.

[90] Headland SE (2015). Neutrophil-derived microvesicles enter cartilage and protect the joint in inflammatory arthritis. Sci. Transl. Med..

[91] Parkos CA (2016). Neutrophil-epithelial interactions: a double-edged sword. Am. J. Pathol..

[92] Tazawa H (2003). Infiltration of neutrophils is required for acquisition of metastatic phenotype of benign murine fibrosarcoma cells: implication of inflammation-associated carcinogenesis and tumor progression. Am. J. Pathol..

[93] Loukinova E (2000). Growth regulated oncogene-alpha expression by murine squamous cell carcinoma promotes tumor growth, metastasis, leukocyte infiltration and angiogenesis by a host CXC receptor-2 dependent mechanism. Oncogene.

[94] Schaider H (2003). Differential response of primary and metastatic melanomas to neutrophils attracted by IL-8. Int. J. Cancer.

[95] Kessenbrock K (2009). Netting neutrophils in autoimmune small-vessel vasculitis. Nat. Med..

[96] Khandpur R (2013). NETs are a source of citrullinated autoantigens and stimulate inflammatory responses in rheumatoid arthritis. Sci. Transl. Med..

[97] Garcia-Romo GS (2011). Netting neutrophils are major inducers of type I IFN production in pediatric systemic lupus erythematosus. Sci. Transl. Med..

[98] Sangaletti S (2012). Neutrophil extracellular traps mediate transfer of cytoplasmic neutrophil antigens to myeloid dendritic cells toward ANCA induction and associated autoimmunity. Blood.

[99] Hu, S. C. S. et al. Neutrophil extracellular trap formation is increased in psoriasis and induces human beta-defensin-2 production in epidermal keratinocytes. *Sci. Rep*. 10.1038/srep31119 (2016). 10.1038/srep31119 PMC4974609 27493143

[100] Guglietta, S. et al. Coagulation induced by C3aR-dependent NETosis drives protumorigenic neutrophils during small intestinal tumorigenesis. *Nat. Commun*. 10.1038/ncomms11037 (2016). 10.1038/ncomms11037 PMC4802169 26996437

[101] Cedervall J (2015). Neutrophil extracellular traps accumulate in peripheral blood vessels and compromise organ function in tumor-bearing animals. Cancer Res..

[102] Fuchs TA (2007). Novel cell death program leads to neutrophil extracellular traps. J. Cell Biol..

[103] Brinkmann V, Zychlinsky A (2007). Beneficial suicide: why neutrophils die to make NETs. Nat. Rev. Microbiol..

[104] Masucci, M. T., Minopoli, M., Del Vecchio, S. & Carriero, M. V. The emerging role of neutrophil extracellular traps (NETs) in tumor progression and metastasis. *Front. Immunol*. 10.3389/fimmu.2020.01749 (2020). 10.3389/fimmu.2020.01749 PMC7524869 33042107

[105] Snoderly, H. T., Boone, B. A. & Bennewitz, M. F. Neutrophil extracellular traps in breast cancer and beyond: current perspectives on NET stimuli, thrombosis and metastasis, and clinical utility for diagnosis and treatment. *Breast Cancer Res*. 10.1186/s13058-019-1237-6 (2019). 10.1186/s13058-019-1237-6 PMC6921561 31852512

[106] Korkmaz B, Moreau T, Gauthier F (2008). Neutrophil elastase, proteinase 3 and cathepsin G: physicochemical properties, activity and physiopathological functions. Biochimie.

[107] Neumann A (2014). The antimicrobial peptide LL-37 facilitates the formation of neutrophil extracellular traps. Biochem. J..

[108] Papayannopoulos V, Metzler KD, Hakkim A, Zychlinsky A (2010). Neutrophil elastase and myeloperoxidase regulate the formation of neutrophil extracellular traps. J. Cell Biol..

[109] Urban, C. F. et al. Neutrophil extracellular traps contain calprotectin, a cytosolic protein complex involved in host defense against Candida albicans. *PLos Pathog*. 10.1371/journal.ppat.1000639 (2009). 10.1371/journal.ppat.1000639 PMC2763347 19876394

[110] Urban CF, Reichard U, Brinkmann V, Zychlinsky A (2006). Neutrophil extracellular traps capture and kill Candida albicans yeast and hyphal forms. Cell. Microbiol..

[111] Villanueva E (2011). Netting neutrophils induce endothelial damage, infiltrate tissues, and expose immunostimulatory molecules in systemic lupus erythematosus. J. Immunol..

[112] Soderberg D (2015). Increased levels of neutrophil extracellular trap remnants in the circulation of patients with small vessel vasculitis, but an inverse correlation to anti-neutrophil cytoplasmic antibodies during remission. Rheumatology.

[113] Martinod K (2016). Neutrophil elastase-deficient mice form neutrophil extracellular traps in an experimental model of deep vein thrombosis. J. Thromb. Haemost..

[114] Nahrendorf M, Swirski FK (2015). Neutrophil-macrophage communication in inflammation and atherosclerosis. Science.

[115] Vitkov L, Klappacher M, Hannig M, Krautgartner WD (2009). Extracellular neutrophil traps in periodontitis. J. Periodontal Res..

[116] Wong SL (2015). Diabetes primes neutrophils to undergo NETosis, which impairs wound healing. Nat. Med..

[117] Yipp BG, Kubes P (2013). NETosis: how vital is it?. Blood.

[118] Behnen M (2014). Immobilized immune complexes induce neutrophil extracellular trap release by human neutrophil granulocytes via Fc gamma RIIIB and Mac-1. J. Immunol..

[119] Gray, R. D. et al. Activation of conventional protein kinase C (PKC) is critical in the generation of human neutrophil extracellular traps. *J. Inflamm*. 10.1186/1476-9255-10-12 (2013). 10.1186/1476-9255-10-12 PMC3643828 23514610

[120] Hakkim A (2011). Activation of the Raf-MEK-ERK pathway is required for neutrophil extracellular trap formation. Nat. Chem. Biol..

[121] Teijeira A (2021). Differential Interleukin-8 thresholds for chemotaxis and netosis in human neutrophils. Eur. J. Immunol..

[122] Cheng Y, Ma XL, Wei YQ, Wei XW (2019). Potential roles and targeted therapy of the CXCLs/CXCR2 axis in cancer and inflammatory diseases. Biochim. Biophys. Acta Rev. Cancer.

[123] Morgan MJ, Liu ZG (2011). Crosstalk of reactive oxygen species and NF-κB signaling. Cell Res..

[124] Neeli, I. & Radic, M. Opposition between PKC isoforms regulates histone deimination and neutrophil extracellular chromatin release. *Front. Immunol*. 10.3389/fimmu.2013.00038 (2013). 10.3389/fimmu.2013.00038 PMC3576869 23430963

[125] Li PX (2010). PAD4 is essential for antibacterial innate immunity mediated by neutrophil extracellular traps. J. Exp. Med..

[126] Thiam HR (2020). NETosis proceeds by cytoskeleton and endomembrane disassembly and PAD4-mediated chromatin decondensation and nuclear envelope rupture. Proc. Natl Acad. Sci. USA.

[127] Pilsczek FH (2010). A novel mechanism of rapid nuclear neutrophil extracellular trap formation in response to Staphylococcus aureus. J. Immunol..

[128] Clark SR (2007). Platelet TLR4 activates neutrophil extracellular traps to ensnare bacteria in septic blood. Nat. Med..

[129] Arpinati L (2020). NETosis in cancer: a critical analysis of the impact of cancer on neutrophil extracellular trap (NET) release in lung cancer patients vs. mice. Cancer Immunol. Immunother..

[130] Li Y (2019). Extracellular RNAs from lung cancer cells activate epithelial cells and induce neutrophil extracellular traps. Int. J. Oncol..

[131] Stehr AM (2022). Neutrophil extracellular traps drive epithelial-mesenchymal transition of human colon cancer. J. Pathol..

[132] Lee W (2019). Neutrophils facilitate ovarian cancer premetastatic niche formation in the omentum. J. Exp. Med..

[133] Telerman, A. et al. Neutrophil extracellular traps are increased in chronic myeloid leukemia and are differentially affected by tyrosine kinase inhibitors. *Cancers*10.3390/cancers14010119 (2022). 10.3390/cancers14010119 PMC8750902 35008283

[134] McInturff AM (2012). Mammalian target of rapamycin regulates neutrophil extracellular trap formation via induction of hypoxia-inducible factor 1 alpha. Blood.

[135] Gupta AK, Hasler P, Holzgreve W, Gebhardt S, Hahn S (2005). Induction of neutrophil extracellular DNA lattices by placental microparticles and IL-8 and their presence in preeclampsia. Hum. Immunol..

[136] Gupta AK (2010). Activated endothelial cells induce neutrophil extracellular traps and are susceptible to NETosis-mediated cell death. FEBS Lett..

[137] Zhang, Y. et al. Interleukin-17-induced neutrophil extracellular traps mediate resistance to checkpoint blockade in pancreatic cancer. *J. Exp. Med*. 10.1084/jem.20190354 (2020). 10.1084/jem.20190354 PMC7953739 32860704

[138] Demers, M. et al. Priming of neutrophils toward NETosis promotes tumor growth. *Oncoimmunology*10.1080/2162402X.2015.1134073 (2016). 10.1080/2162402X.2015.1134073 PMC4910712 27467952

[139] Mao, Z. et al. CXCL5 promotes gastric cancer metastasis by inducing epithelial-mesenchymal transition and activating neutrophils. *Oncogenesis*10.1038/s41389-020-00249-z (2020). 10.1038/s41389-020-00249-z PMC7338464 32632106

[140] Verbeke H (2011). Isotypic neutralizing antibodies against mouse GCP-2/CXCL6 inhibit melanoma growth and metastasis. Cancer Lett..

[141] Barbu, E. A., Mendelsohn, L., Samsel, L. & Thein, S. L. Pro-inflammatory cytokines associate with NETosis during sickle cell vaso-occlusive crises. *Cytokine*10.1016/j.cyto.2019.154933 (2020). 10.1016/j.cyto.2019.154933 PMC8419744 31778959

[142] Keshari, R. S. et al. Cytokines induced neutrophil extracellular traps formation: implication for the inflammatory disease condition. *PLoS One*10.1371/journal.pone.0048111 (2012). 10.1371/journal.pone.0048111 PMC3482178 23110185

[143] Tatsiy, O. & McDonald, P. P. Physiological stimuli induce PAD4-dependent, ROS-independent NETosis, with early and late events controlled by discrete signaling pathways. *Front. Immunol*. 10.3389/fimmu.2018.02036 (2018). 10.3389/fimmu.2018.02036 PMC6153332 30279690

[144] Cedervall J, Zhang YY, Olsson AK (2016). Tumor-induced NETosis as a risk factor for metastasis and organ failure. Cancer Res..

[145] Leal, A. C. et al. Tumor-derived exosomes induce the formation of neutrophil extracellular traps: Implications for the establishment of cancer-associated thrombosis. *Sci. Rep*. 10.1038/s41598-017-06893-7 (2017). 10.1038/s41598-017-06893-7 PMC5526939 28743887

[146] Oklu R, Sheth RA, Wong KHK, Jahromi AH, Albadawi H (2017). Neutrophil extracellular traps are increased in cancer patients but does not associate with venous thrombosis. Cardiovasc. Diagn. Ther..

[147] Richardson, J. J. R., Hendrickse, C., Gao-Smith, F. & Thickett, D. R. Neutrophil extracellular trap production in patients with colorectal cancer in vitro. *Int. J. Inflam*. 10.1155/2017/4915062 (2017). 10.1155/2017/4915062 PMC5554570 28828191

[148] Yang LB (2020). DNA of neutrophil extracellular traps promotes cancer metastasis via CCDC25. Nature.

[149] Kaltenmeier CT (2021). Neutrophil extracellular traps as a novel biomarker to predict recurrence-free and overall survival in patients with primary hepatic malignancies. HPB.

[150] Kim SJ (2020). Validation of CDr15 as a new dye for detecting neutrophil extracellular trap. Biochem. Bioph. Res. Commun..

[151] Rosinski M, Yarmush ML, Berthiaume F (2004). Quantitative dynamics of in vivo bone marrow neutrophil production and egress in response to injury and infection. Ann. Biomed. Eng..

[152] Shijo H (1998). Evaluation of neutrophil functions after experimental abdominal surgical trauma. Inflamm. Res..

[153] Boettcher S (2014). Endothelial cells translate pathogen signals into G-CSF-driven emergency granulopoiesis. Blood.

[154] Woytschak J (2016). Type 2 interleukin-4 receptor signaling in neutrophils antagonizes their expansion and migration during Infection and Inflammation. Immunity.

[155] Zhang Q (2010). Circulating mitochondrial DAMPs cause inflammatory responses to injury. Nature.

[156] Visser T, Hietbrink F, Groeneveld KM, Koenderman L, Leenen LP (2011). Isolated blunt chest injury leads to transient activation of circulating neutrophils. Eur. J. Trauma Emer. Surg..

[157] Olutoye OO, Zhu X, Cass DL, Smith CW (2005). Neutrophil recruitment by fetal porcine endothelial cells: implications in scarless fetal wound healing. Pediatr. Res..

[158] Orlova VV (2007). A novel pathway of HMGB1-mediated inflammatory cell recruitment that requires Mac-1-integrin. EMBO J..

[159] Wang Y, Du F, Hawez A, Morgelin M, Thorlacius H (2019). Neutrophil extracellular trap-microparticle complexes trigger neutrophil recruitment via high-mobility group protein 1 (HMGB1)-toll-like receptors(TLR2)/TLR4 signalling. Br. J. Pharmacol..

[160] Huang C, Niethammer P (2018). Tissue damage signaling is a prerequisite for protective neutrophil recruitment to microbial infection in zebrafish. Immunity.

[161] Wheeler, D. S. et al. Extracellular Hsp72, an endogenous DAMP, is released by virally infected airway epithelial cells and activates neutrophils via Toll-like receptor (TLR)-4. *Respir. Res*. 10.1186/1465-9921-10-31 (2009). 10.1186/1465-9921-10-31 PMC2679007 19405961

[162] Pouwels SD (2017). Increased neutrophil expression of pattern recognition receptors during COPD exacerbations. Respirology.

[163] Hanahan D, Coussens LM (2012). Accessories to the crime: functions of cells recruited to the tumor microenvironment. Cancer cell.

[164] Chou RC (2010). Lipid-cytokine-chemokine cascade drives neutrophil recruitment in a murine model of inflammatory arthritis. Immunity.

[165] Elliott MR (2009). Nucleotides released by apoptotic cells act as a find-me signal to promote phagocytic clearance. Nature.

[166] Esmann L (2010). Phagocytosis of apoptotic cells by neutrophil granulocytes: diminished proinflammatory neutrophil functions in the presence of apoptotic cells. J. Immunol..

[167] Lazaro-Diez, M. et al. Human neutrophils phagocytose and kill Acinetobacter baumannii and A. pittii. *Sci. Rep*. 10.1038/s41598-017-04870-8 (2017). 10.1038/s41598-017-04870-8 PMC5496873 28676640

[168] Simpson DM, Ross R (1972). The neutrophilic leukocyte in wound repair a study with antineutrophil serum. J. Clin. Investig..

[169] Nishio N, Okawa Y, Sakurai H, Isobe K (2008). Neutrophil depletion delays wound repair in aged mice. Age.

[170] Tecchio C, Cassatella MA (2014). Neutrophil-derived cytokines involved in physiological and pathological angiogenesis. Chem. Immunol. Allergy.

[171] Aldabbous L (2016). Neutrophil extracellular traps promote angiogenesis: evidence from vascular pathology in pulmonary hypertension. Arterioscler. Thromb. Vasc. Biol..

[172] Yipp BG (2012). Infection-induced NETosis is a dynamic process involving neutrophil multitasking in vivo. Nat. Med..

[173] van Dam LS, Rabelink TJ, van Kooten C, Teng YKO (2019). Clinical implications of excessive neutrophil extracellular trap formation in renal autoimmune diseases. Kidney Int. Rep..

[174] Stakos D, Skendros P, Konstantinides S, Ritis K (2020). Traps N’ clots: NET-mediated thrombosis and related diseases. Thromb. Haemost..

[175] von Meijenfeldt FA (2018). Elevated plasma levels of cell-free DNA during liver transplantation are associated with activation of coagulation. Liver Ttranspl..

[176] Paunel-Gorgulu, A. et al. cfDNA correlates with endothelial damage after cardiac surgery with prolonged cardiopulmonary bypass and amplifies NETosis in an intracellular TLR9-independent manner. *Sci. Rep*. 10.1038/s41598-017-17561-1 (2017). 10.1038/s41598-017-17561-1 PMC5727170 29234042

[177] Banki F (2007). Plasma DNA as a molecular marker for completeness of resection and recurrent disease in patients with esophageal cancer. Arch. Surg. Chic..

[178] Ludovini V (2008). Plasma DNA, microsatellite alterations, and p53 tumor mutations are associated with disease-free survival in radically resected non-small cell lung cancer patients: a study of the perugia multidisciplinary team for thoracic oncology. J. Thorac. Oncol..

[179] Decker, A. S. et al. Prognostic role of blood NETosis in the progression of head and neck cancer. *Cells*10.3390/cells8090946 (2019). 10.3390/cells8090946 PMC6770876 31438586

[180] Park J (2016). Cancer cells induce metastasis-supporting neutrophil extracellular DNA traps. Sci. Transl. Med..

[181] Thalin, C. et al. Citrullinated histone H3 as a novel prognostic blood marker in patients with advanced cancer. *Plos One*10.1371/journal.pone.0191231 (2018). 10.1371/journal.pone.0191231 PMC5764486 29324871

[182] Khan, U. et al. Neutrophil extracellular traps in colorectal cancer progression and metastasis. *Int. J. Mol. Sci*. 10.3390/ijms22147260 (2021). 10.3390/ijms22147260 PMC8307027 34298878

[183] Estrella V (2013). Acidity generated by the tumor microenvironment drives local invasion. Cancer Res..

[184] Seliger B (2005). Strategies of tumor immune evasion. BioDrug.

[185] Zavyalova MV (2019). Intravasation as a key step in cancer metastasis. Biochemistry.

[186] Desgrosellier JS (2009). An integrin alpha(v)beta(3)-c-Src oncogenic unit promotes anchorage-independence and tumor progression. Nat. Med..

[187] Strilic B, Offermanns S (2017). Intravascular survival and extravasation of tumor cells. Cancer cell.

[188] Phan TG, Croucher PI (2020). The dormant cancer cell life cycle. Nat. Rev. Cancer.

[189] Parhi, L. et al. Breast cancer colonization by Fusobacterium nucleatum accelerates tumor growth and metastatic progression. *Nat. Commun*. 10.1038/s41467-020-16967-2 (2020). 10.1038/s41467-020-16967-2 PMC7320135 32591509

[190] Cools-Lartigue J, Spicer J, Najmeh S, Ferri L (2014). Neutrophil extracellular traps in cancer progression. Cell. Mol. Life Sci..

[191] Das, A., Monteiro, M., Barai, A., Kumar, S. & Sen, S. MMP proteolytic activity regulates cancer invasiveness by modulating integrins. *Sci. Rep*. 10.1038/s41598-017-14340-w (2017). 10.1038/s41598-017-14340-w PMC5660204 29079818

[192] Liotta LA (2016). Adhere, degrade, and move: The three-step model of invasion. Cancer Res..

[193] Deryugina, E. et al. Neutrophil elastase facilitates tumor cell intravasation and early metastatic events. *iScience*10.1016/j.isci.2020.101799 (2020). 10.1016/j.isci.2020.101799 PMC7702017 33299970

[194] Bjork J, Hedqvist P, Arfors KE (1982). Increase in vascular permeability induced by leukotriene B4 and the role of polymorphonuclear leukocytes. Inflammation.

[195] Cools-Lartigue J (2013). Neutrophil extracellular traps sequester circulating tumor cells and promote metastasis. J. Clin. Investig..

[196] Najmeh S (2017). Neutrophil extracellular traps sequester circulating tumor cells via beta1-integrin mediated interactions. Int. J. Cancer.

[197] Park SY, Nam JS (2020). The force awakens: metastatic dormant cancer cells. Exp. Mol. Med..

[198] Albrengues, J. et al. Neutrophil extracellular traps produced during inflammation awaken dormant cancer cells in mice. *Science*10.1126/science.aao4227 (2018). 10.1126/science.aao4227 PMC6777850 30262472

